# Fatigue Behaviour of PA66 GF30 at Different Temperatures

**DOI:** 10.3390/polym17010042

**Published:** 2024-12-27

**Authors:** Marko Zadravec, Janez Kramberger, Branko Nečemer, Srečko Glodež

**Affiliations:** Faculty of Mechanical Engineering, University of Maribor, Smetanova 17, 2000 Maribor, Slovenia; marko.zadravec3@student.um.si (M.Z.); janez.kramberger@um.si (J.K.); branko.necemer@um.si (B.N.)

**Keywords:** PA66 GF30, increased temperatures, fatigue, experimental testing

## Abstract

A comprehensive experimental investigation to understand the mechanical properties and fatigue behaviour of glass-reinforced polyamide (PA66 GF30) at different temperatures is presented in this paper. The specimens for quasi-static and fatigue testing were machined from previously extruded plates, where two orientations were considered: (i) the extrusion direction (ED) and (ii) the direction perpendicular to extrusion (PED). Both the quasi-static and fatigue tests were performed under different temperatures (22 °C and 100 °C). The fatigue tests were performed in a load control regime under pulsating loading (*R* = 0.1) to create S–N curves for all the temperatures and loading directions. The experimental results of the quasi-static tests showed that the test specimens manufactured in the extrusion direction have better mechanical properties when compared to those of the specimens manufactured perpendicular to the extrusion direction. Furthermore, the analysis of the quasi-static tensile test results showed that tensile strength, yield strength, and the modulus of elasticity are significantly dependent on the temperature and deteriorate when the temperature is increased from 22 °C to 100 °C. The results of the fatigue tests showed that at both the temperatures (22 °C and 100 °C), the samples produced in the direction of extrusion exhibited higher fatigue strength than those produced perpendicular to the direction of extrusion. For all the sample orientations, the fatigue strength decreased significantly with increasing temperature. The obtained experimental results could be very useful when designing and dimensioning different dynamically loaded engineering components made of PA66 GF30 subjected to high temperatures.

## 1. Introduction

Polymers and polymeric materials are indispensable in almost all areas of the economy today. Due to their versatile properties, polymers are used in agriculture for crop protection and to improve the effectiveness of pesticides and herbicides [1], in medicine [2] and pharmacy [3], in energy generation and storage [4], in water treatment technology [5], etc. Good mechanical properties, high dynamic strength, and corrosion resistance make polymeric composites indispensable for the automotive [6,7,8], aerospace [9,10,11], and military industries [12,13,14].

Polymeric materials, such as polyolefins, polyurethanes, and polyamides, are well suited to large-scale production in the automotive industry due to their low specific weight, design flexibility, and cost efficiency [15,16,17,18,19]. This is reflected in the fact that cars today consist of, on average, 222 kg of different plastic parts, which corresponds to 17.7% of the weight of an average passenger car [20]. Polymeric parts can be found in the exterior of a vehicle, in the interior, and under the bonnet. However, in order to reduce the weight of vehicles, it is possible to combine polymer materials with metals, especially where the parts are exposed to higher loads [21,22].

The current driving force behind developments in the automotive industry is the need to reduce emissions and fuel consumption, which can be achieved by reducing vehicles’ weight. This can be achieved using innovative manufacturing techniques for producing components and using polyamide-based materials to replace the conventional metallic materials [8]. Due to their excellent mechanical and thermal properties, PA6, PA66, and PA12 are the most commonly used polyamide materials [23]. However, the properties of these materials can be improved by adding reinforcing fibres, either glass or carbon. The direct comparison of the mechanical properties of reinforced and unreinforced materials show that adding fibres increases failure stress and the modulus of elasticity, but significantly reduces failure strain, indicating that the fibres significantly increase the material’s stiffness [24,25,26,27,28,29]. As the proportion of glass fibres in the polyamide matrix increases, the modulus and strength increase linearly and reach a maximum between 40 and 50% fibre, while the impact strength of the material initially decreases at low fibre contents and reaches a minimum at 4%, after which it increases again [27]. The orientation and diameter of reinforcing fibres also have an important influence on the mechanical properties. The highest tensile strength and modulus can be achieved when the fibres are oriented in the direction of tensile stress. However, as the fibre diameter increases, the tensile strength and elongation at break decrease, and the tensile modulus increases as a larger fibre diameter leads to a higher stiffness [30].

Temperature is a significant factor in dimensioning components made of polyamide materials and glass fibre-reinforced polyamide composites. Composites such as PA66 GF30 are suitable for high temperatures, but great care must be taken as the mechanical properties change significantly with temperature. Costa Mattos et al. [31] have developed an algebraic equation to produce a stress–strain curve of polyamides (PA11, PA12, and PA66 GF30) at different temperatures, such as the result of the ASTM D638-14 test. The components in the automotive industry that are made of glass fibre-reinforced composites are, in most cases, exposed to vibration fatigue. Therefore, in addition to the aforementioned effects on mechanical properties, knowledge of the vibration behaviour of components and materials is also crucial for reliable component design. In recent years, much research has been carried out on the vibration fatigue of the composite material PA66 GF and on the mechanism of crack growth. Chebbi et al. [32] found that increasing the proportion of short glass fibres in PA66 has no influence on the kinetics of crack growth, but does have an influence on the service life. As the fibre content increases, the service life and the associated slope of the S-N curve decreases. The frequency and the direction of loading also play important roles, as the heating effect and associated additional thermal fatigue occur at high frequencies [33]. Vibration fatigue at temperatures above the glass transition temperature of the matrix leads to a reduction in fatigue strength due to increased ductility of the matrix. Considering this fact, Brunbauer et al. [34] found that the stress amplitudes of a standard glass fibre sample made of PA66 GF35 are greater with an alternating load of *R* = −1 than with a pulsating tensile load of *R* = 0.1. When designing components made of composite materials, the influence of moisture on the mechanical properties and vibration fatigue of components must not be ignored. Exposure to moisture reduces tensile strength and the modulus as adhesion between the reinforcing fibres and the polymer matrix deteriorates [35]. However, the elongation at break is significantly increased as water molecules reduce the strength of the hydrogen bonds formed between the polyamide in the matrix.

Many factors influence the mechanical properties of glass fibre-reinforced polyamides, and the choice of material, the manufacturing process, and the dimensioning of products made from these materials can be very time-consuming. The most significant problem is that the fibres may be unevenly distributed across the cross-section, reducing their reinforcing effect. In this article, a simple extruded sheet of PA66 GF30 is used to show how the mechanical properties change depending on the direction of extrusion and how the influence of elevated temperatures deteriorates the mechanical properties of the materials. The PA66 GF30 material was selected because it is suitable for use at high temperatures and can therefore be used to manufacture secondary engine components as it is resistant to a wide range of oils, greases, and fuels. The secondary components typically found in engines are the components of the engine’s lubrication system that are exposed to elevated temperatures due to the heating of engine oil. During regular engine operation, the oil temperatures reach a temperature around 100 °C [36]. The components of the lubrication system must therefore be able to withstand these temperatures. According to the values specified by extrusion manufacturers, PA66 GF30 can be exposed to an elevated temperature of 180 °C for short periods and 110 °C for long periods and has a melting temperature of 254 °C (see Table 1). When dimensioning the engineering components (i.e., secondary components of combustion engines) subjected to dynamic loads and elevated temperatures, it is crucial to obtain the fatigue life of such components. It is also essential to know how the extrusion direction of the sheets, which affects the orientation of the reinforcing fibres, can influence the mechanical properties and shorten the duration of exposure to elevated temperatures. Therefore, the main objective of the research is to obtain S-N curves of the PA66 GF30 material that can be used in future studies to calculate the lifetime of components made of this material at elevated temperatures.

This work investigates the mechanical characteristics and fatigue behaviour of thin-walled specimens with a cross-sectional area of 9 mm^2^ made from glass fibre-reinforced composite material PA66 GF30. The test specimens were cut from an extruded plate so that the influence of fibre orientation on the mechanical properties and fatigue life could also be investigated. Quasi-static tests and fatigue tests were carried out at room temperature (22 °C) and an elevated temperature (100 °C) using an electrodynamic testing machine Zwick/Roel LTM3. The obtained experimental results can be further used as primary input data for the subsequent computational analyses of structural parts made of glass fibre-reinforced composite material PA66 GF30.

## 2. Materials and Methods

### 2.1. Material and Geometry of Specimens

**Figure 1:**
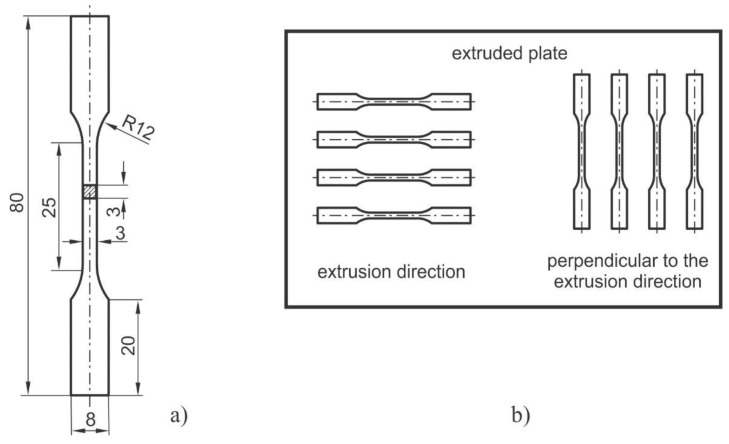
The geometry of the specimen (**a**) and the preparation of the specimens (**b**).

The proposed research work assumed glass fibre-reinforced polymer PA66 GF30 as the base material. The basic mechanical, thermal, and physical properties are listed in Table 1. The specimens (see Figure 1a) that underwent subsequent experimental testing were manufactured with a milling process from an extruded plate made of PA66 GF 30, where two orientations were considered (see Figure 1b): (i) the extrusion direction (ED specimens) and (ii) the direction perpendicular to extrusion (PED specimens).

All the manufactured specimens were checked for critical cross-sectional geometry deviations from their theoretical geometry, as defined in Figure 1. Table 2 contains average values for width *t_a_* and thickness *t_b_* and the standard deviation for the specimens produced in the extrusion direction (ED specimens) and perpendicular to the extrusion direction (PED specimens).

### 2.2. Experimental Testing

**Figure 2:**
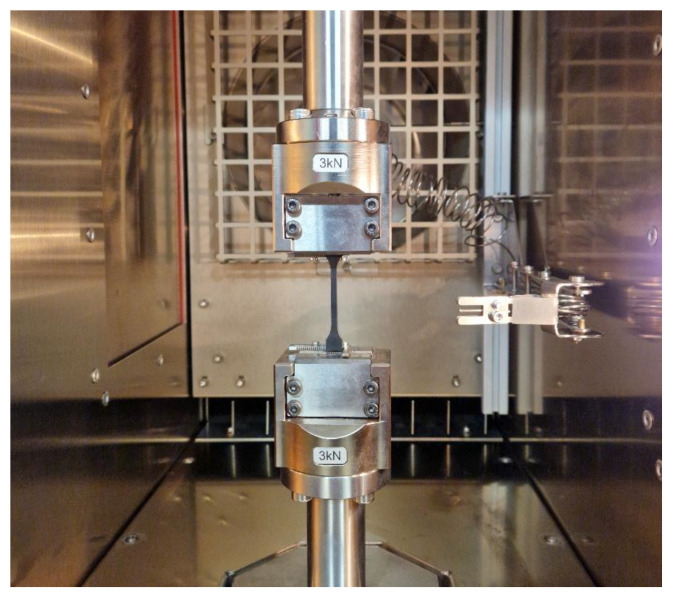
The specimen mounted onto the temperature chamber Zwick/Roel.

Quasi-static and fatigue tests were performed with a Zwick/Roel GmbH & Co. KG, Ulm, Germany Electrodynamic Testing Machine LTM3 under different temperatures (22 °C and 100 °C) using the Zwick/Roel temperature chamber (see Figure 2). Force was monitored with a 3 kN tensile–compressive load cell mounted on the testing machine. At the same time, strain was controlled on the entire length between clamping jaws. For the quasi-static tests, eight samples were tested for both the extrusion direction (ED specimens) and perpendicular to the extrusion direction (PED specimens) under a load rate of 2.5 mm/min.

Fatigue tests were performed at room and elevated temperatures (22 °C and 100 °C) for both specimen orientations. The fatigue tests were performed under stress control at a load ratio *R* = 0.1. Due to the alternating load, care was taken to ensure that the specimens did not heat up during the tests. Based on this assumption, the loading frequency was set to 2 Hz. The maximum amplitude stress at which the specimens were tested was 27 MPa, and the minimum amplitude stress was 15 MPa. Both quasi-static and fatigue tests were performed under controlled climatic conditions with a constant ambient temperature and constant humidity.

## 3. Results and Discussion

### 3.1. Quasi-Static Tests

**Figure 3:**
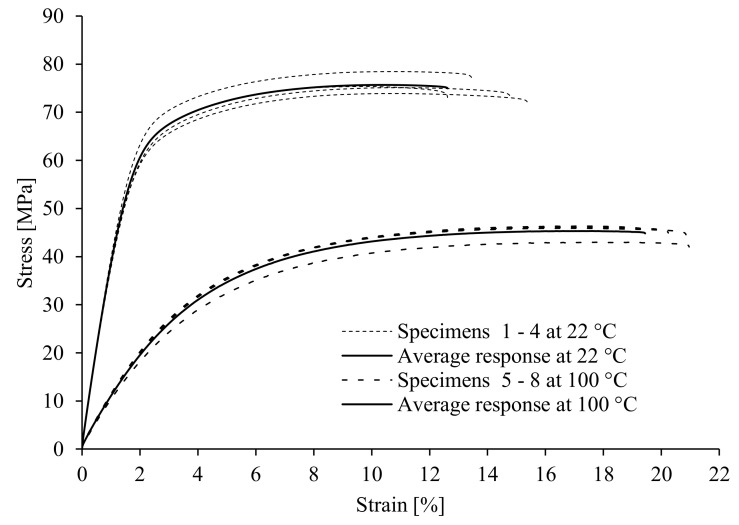
Stress–strain curves for PED specimens.

**Figure 4:**
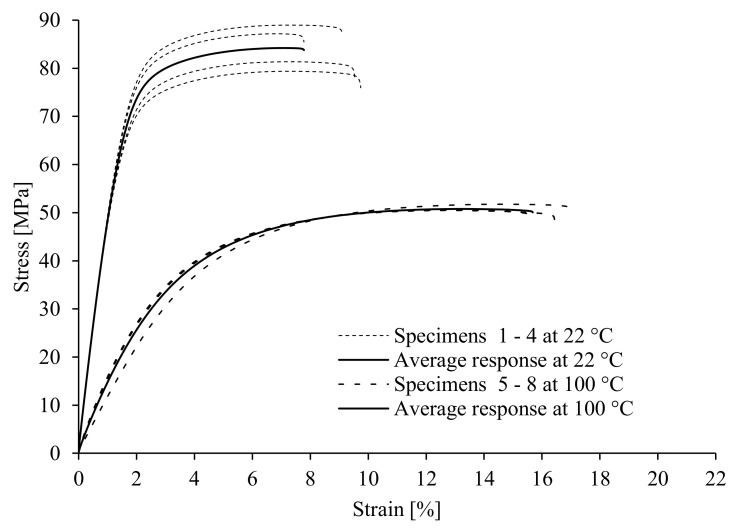
Stress–strain curves for ED specimens.

Figure 3 shows the stress–strain curves for PED specimens at room temperature (22 °C) and an elevated temperature (100 °C). The analysis of the quasi-static tensile test results shows a relatively good correlation between the tensile strength and the modulus of elasticity at room temperature with the values found in the literature and listed in Table 1. However, the mechanical properties (tensile strength, yield stress, and Young’s modulus) change significantly with temperature. As the temperature increases, the strength parameters (tensile strength, yield stress, and Young’s modulus) decrease considerably. On the other hand, the elongation at break is bigger at an elevated temperature. Similar conclusions can also be made for the ED specimens (see Figure 4). The characteristic values of mechanical properties from Figure 3 and Figure 4 are summarised in Table 3.

Possibly, the main reason for the significant deterioration in mechanical properties with increasing temperature is the reduction in stiffness of the matrix, as the amorphous parts become more mobile above the glass transition temperature, which is 48 °C for the polymer used in this study (see Table 1). In addition, an increase in temperature weakens the intermolecular hydrogen bonds between the amide groups within the polyamide matrix. At higher temperatures, thermomechanical forces occur at the interface between the matrix and the fibres due to the different coefficients of thermal expansion, which lead to a deterioration in adhesion forces between the matrix and the fibres. At the same time, the hydrolysis of the polyamide molecules can be accelerated at higher temperatures due to the presence of moisture.

The experimental results of quasi-static tests (see Table 3) show that the test specimens manufactured in the extrusion direction (ED specimens) have better mechanical properties if compared to those of the specimens manufactured perpendicular to the extrusion direction (PED specimens). However, the elongation at break is slightly smaller for the ED specimens than for the PED specimens.

The most significant scattering of experimental results was found for the ED samples at room temperature (see Figure 4). This is probably due to the fact that the percentage of reinforcing fibres in the critical cross-section varied from sample to sample. The extrusion technique can influence fibres’ distribution and orientation, so great care must be taken when evaluating the mechanical properties of materials and dimensioning components made from these materials. Even with a simple sheet used for the samples in our case, we cannot observe the constant material properties between the samples in the same direction at room temperature. It can be concluded from the results that the extrusion process for large sheets offers less control over fibre distribution in the cross-section of the sheets. Although the fibres are predominantly oriented in the direction of material flow or the extrusion direction, they are not evenly distributed, which becomes even more evident when such sheets are processed into thin-walled test specimens, as conducted in this study. The fibre distribution across the cross-section of the extruded sheets alone could probably be improved by extruding smaller sheets or by injection moulding the test specimens. At higher temperatures, however, the tensile test results were less scattered, as the stiffness of the material matrix deteriorated significantly and therefore the influence of the proportion of reinforcing fibres in the critical cross-section on the scattering is lower. A deviation in the result can also be observed in the samples produced perpendicular to the extrusion direction (see Figure 3), but it is less obvious than that of the samples produced in the extrusion direction. This is because the reinforcing function of the fibres in the samples perpendicular to the extrusion direction is worse than that of the samples in the extrusion direction. This means that the proportion of fibres in the critical cross-section has less of an influence on the mechanical properties.

**Figure 5:**
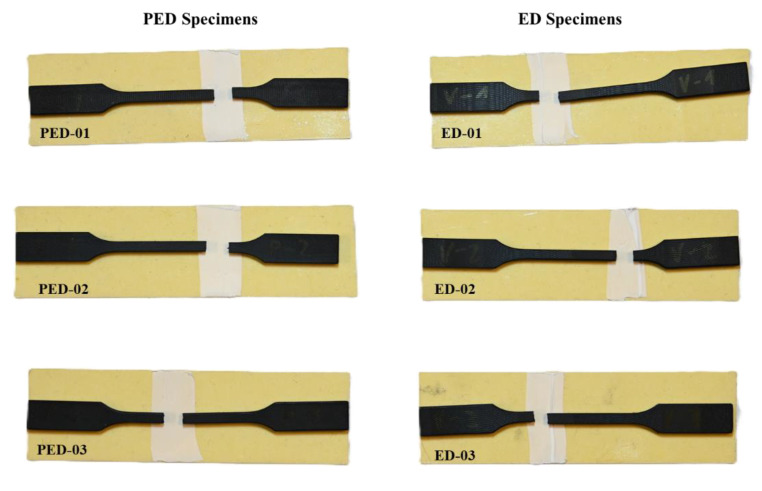
PED specimens and ED specimens after quasi-static tensile test at 22 °C.

Figure 5 shows the fractured PED and ED specimens after the quasi-static tensile test. Three PED fracture specimens and three ED specimens tested at a room temperature of 22 °C are shown. The fracture surfaces of the samples tested at elevated temperatures were similar to the fracture surfaces of the samples tested at room temperature.

### 3.2. Fatigue Tests

**Figure 6:**
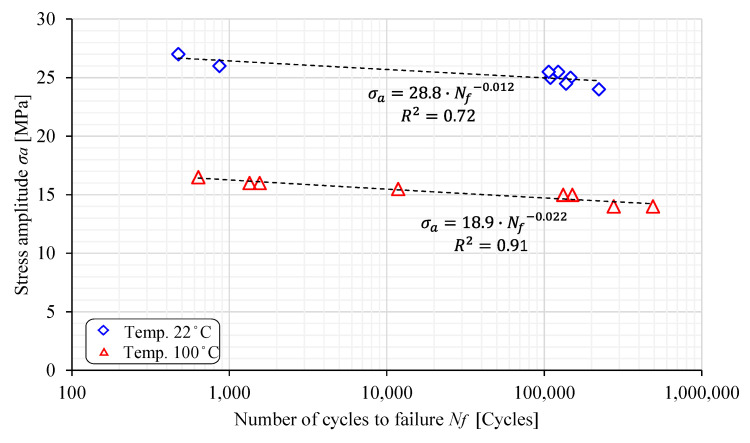
The S-N curves for the PED specimens.

**Figure 7:**
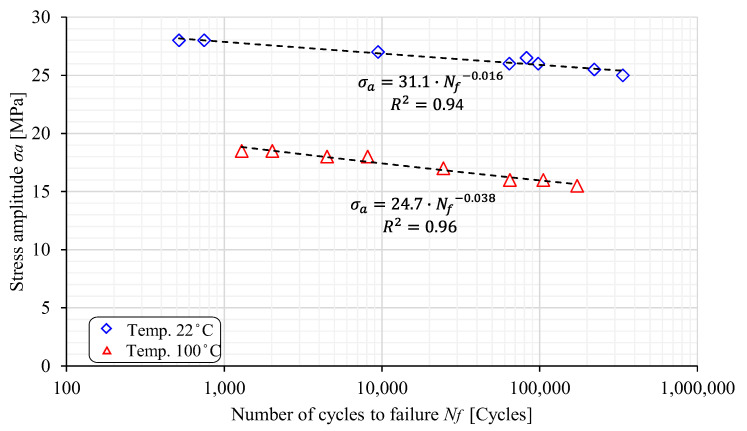
The S-N curves for the ED specimens.

High-cycle fatigue (HCF) tests for both the PED and ED specimens were performed for six different amplitude stresses ranging between 15 MPa and 27 MPa and two different temperatures (22 °C and 100 °C). The experimental results are shown in Figure 6 and Figure 7, where the S-N curves are plotted in the diagram σ_a_ − log*N_f_*. The curves can be expressed mathematically with the following equation:

(1) $$\sigma_{a}=C_{1}N_{f}^{C_{2}}$$

where $\sigma_{a}$ is the amplitude stress; $N_{f}$ is the number of cycles until failure; and $C_{1}$ and $C_{2}$ are the material constants, respectively. It is clear from Figure 5 that the most extensive scattering of experimental results was found for the PED specimens at 22 °C, where the coefficient of determination was only $R^{2}=0.72$. This is probably due to the fact that the PED specimens were produced perpendicular to the extrusion direction, which reduces the stiffening function of the glass fibres in the base material. At an increased temperature of 100 °C, however, the scattering of the results was lower because the polyamide matrix has poorer mechanical properties, and consequently the orientation and quantity of fibres in the specimens have less of an influence. The experimental results were significantly less scattered for the ED specimens, which is valid for both the considered temperatures (22 °C and 100 °C). The complete results for the material constant are summarised in Table 4.

**Figure 8:**
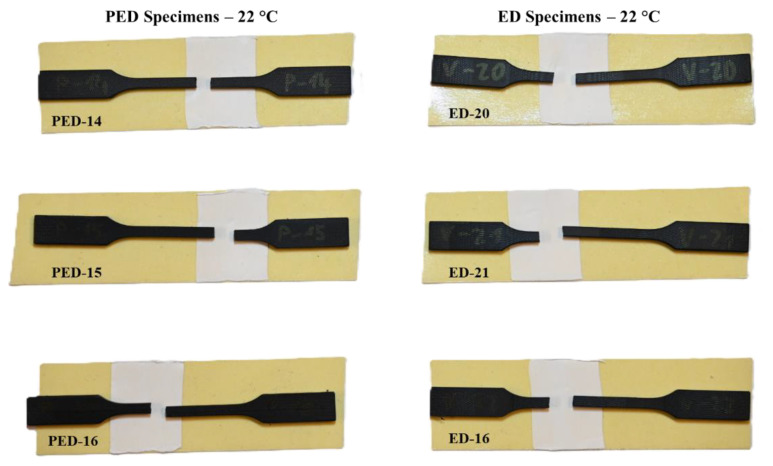
Fatigue fractures of PED and ED specimens at 22 °C.

**Figure 9:**
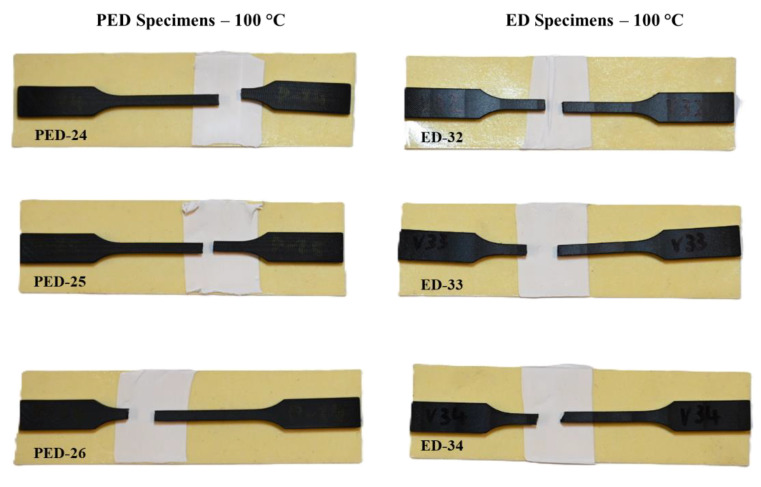
Fatigue fractures of PED and ED specimens at 100 °C.

Figure 8 and Figure 9 show the fractured PED and ED specimens that were subjected to fatigue loading at a room temperature of 22 °C and elevated temperatures of 100 °C. All the figures demonstrate a typical brittle fracture, as it also appeared by quasi-static loading (see Figure 5).

**Figure 10:**
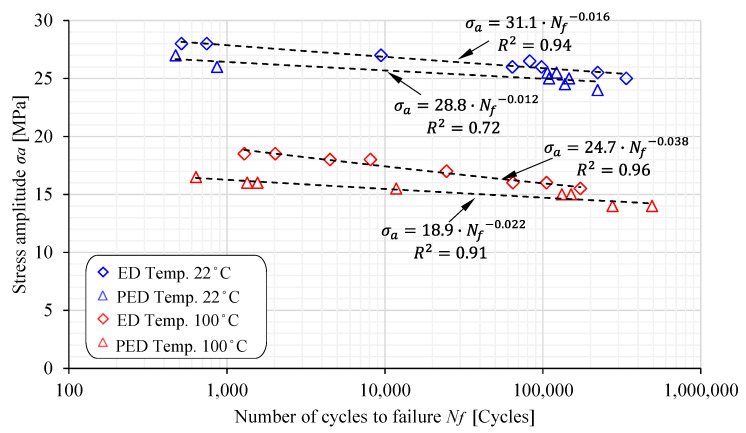
Comparison of S-N curves for PED and ED specimens.

Figure 10 summarises all the experimental results taken during the fatigue test of the PED and ED samples at room and elevated temperatures. For each sample type and temperature, we were able to plot S-N curves for the PA66 GF30 material with only six samples. Due to the low loading frequency of 2 Hz, which avoids the risk of heating the samples during the test, and thus the risk of creep occurring, we significantly increased the test duration. Performing a large number of measurements is very time-consuming, but in our case we were able to determine S-N curves for a specific specimen type and at different temperatures with sufficiently good reliability.

As can be seen from Figure 10, an increase in temperature above the glass transition temperature changes the shape of the S-N curves considerably. The slope of the curves increases, as evidenced by the decrease in the material coefficient $C_{2}$ for both the sample types ED and PED. The material coefficient $C_{1}$ was also reduced. It is also interesting to note that the difference between the material coefficients $C_{1}$ and $C_{2}$ at room temperature for the PED and ED samples is smaller than the difference at the elevated temperature. The results show that at 10,000 cycles, the amplitude stress of the ED samples decreases from 25.9 MPa to only 15.9 MPa, while the amplitude stress of the PED samples decreases from 25.1 MPa to 14.7 MPa. This decrease is somewhat more significant for the PED samples and amounts to 10.4 MPa. When dimensioning dynamically loaded engineering components made of PA66 GF30, we need to know the operating conditions, the loads, and the material properties to accurately estimate the component’s service life, as the temperature rise and fibre orientation significantly influence the service life.

## 4. Conclusions

In the present study, quasi-static and high-cycle fatigue tests were performed on test specimens made of glass fibre-reinforced polyamide composite PA66 GF30 at a room temperature of 22 °C and an elevated temperature of 100 °C. The specimens were cut from an extruded plate to investigate how the external loading regarding the extrusion direction affects the specimen’s mechanical properties and fatigue behaviour. Based on the obtained experimental results and their evaluation, the following conclusions can be made:

- The results of quasi-static tests showed that mechanical properties (tensile strength, yield stress, and Young’s modulus) change significantly with temperature. As the temperature increases (from 22 °C to 100 °C), the strength parameters (tensile strength, yield stress, and Young’s modulus) decrease considerably. Furthermore, the experimental results of the quasi-static tests also showed that the test specimens manufactured in the extrusion direction (ED specimens) have better mechanical properties than those manufactured perpendicular to the extrusion direction (PED specimens). The greatest scatter in the results was found for the specimens produced in the extrusion direction at a room temperature of 22 °C. The most likely reason for widespread scattering is that the reinforcing glass fibres are unevenly distributed in the critical cross-section of the samples. However, the experimental results of the quasi-static tensile test at room conditions (tensile strength and modulus of elasticity) showed a relatively good agreement if compared to the available results in the literature and stated by the material manufacturers. The greatest difference was found in the elongation at break for the samples produced in the extrusion direction.
- The results of fatigue tests showed that for both the considered temperatures (22 °C and 100 °C), the test specimens manufactured in the extrusion direction (ED specimens) demonstrated better fatigue resistance than those manufactured perpendicular to the extrusion direction (PED specimens). For both the specimen’s orientations, fatigue resistance reduced significantly with temperature.
- The results obtained in this study provide a reasonable basis for the dimensioning of products and components made of PA66 GF30 at room or elevated temperatures.
- Further investigation should also focus on the microstructure of analysed material (i.e., length and diameter of glass fibres and their distribution in the base material), which may affect the mechanical and fatigue properties.
- According to the supplier of the extruded sheets used to manufacture the samples in this study, PA66 GF30 can withstand a short exposure time at elevated temperatures of 180 °C. Based on this assumption, future studies will focus on the short-life fatigue behaviour of PA66 GF30. Furthermore, it would also be essential to investigate the interaction between elevated temperatures and the presence of a medium, such as water or other fluids, on the fatigue life of the analysed component.

## Figures and Tables

**Table 1:** Basic mechanical, thermal, and physical properties of analysed polymer PA66 GF30 (adapted from [37], Ensinger, 2024, [38,39,40,41,42]).

**Mechanical properties**		
Tensile strength	ISO 527-2	91 MPa
Elongation at break	ISO 527-2	14%
Tensile *E*-modulus	ISO 527-2	5500 MPa
Charpy impact strength	ISO 179-1	75 kJ/m^2^
**Thermal properties**		
Glass transition temperature	ISO 11357	+48 °C
Melting temperature	ISO 11357	+254 °C
max. Service temperature (long term)		+110 °C
max. Service temperature (short term)		+180 °C
**Other properties**		
Density		1.34 g/cm^3^
Moisture absorption at saturation (+23 °C)	ISO 62	2.0%
Shore hardness	ISO 868	86

**Table 2:** Average values $\bar{x}$ and standard deviations σ of width and thickness of specimens produced in extrusion direction (ED specimens) and perpendicular to extrusion direction (PED specimens).

	ED Specimens		PED Specimens	
	$\bar{x}$	σ	$\bar{x}$	σ
Width $t_{a}$ [mm]	2.96	0.02	2.96	0.02
Thickness $t_{b}\;[mm]$	3.08	0.01	3.06	0.01

**Table 3:** Mechanical properties PA66 GF30 for both loading orientations.

	PED Specimens		ED Specimens	
Testing temperature [°C]	22	100	22	100
Young’s modulus *E* [MPa]	4000	1040	5705	1380
Yield stress *R_e_* [MPa]	67.7 *	35.1 **	77.3 *	40.9 **
Tensile strength *R_m_* [MPa]	75.7	45.3	84.2	50.8
Elongation at break [%]	13.4	21	8.5	17

* Evaluated at 1.5% strain; ** evaluated at 2% strain.

**Table 4:** Material constants *C*_1_ and *C*_2_.

	PED Specimens		ED Specimens	
Testing temperature [°C]	22	100	22	100
Constant *C*_1_ [MPa]	28.8	18.9	31.1	24.7
Constant *C*_2_ [/]	−0.012	−0.022	−0.016	−0.038

## Data Availability

The original contributions presented in the study are included in the article, further inquiries can be directed to the corresponding author.
